# Impact of Infertility and Medically Assisted Reproduction Treatments on Female Sexuality

**DOI:** 10.5935/1518-0557.20240100

**Published:** 2025

**Authors:** Badra Bannour, Darine Salem, Rania Bannour, Omar Khalil Ben Saad, Imen Bannour

**Affiliations:** 1 Obstetrics and Gynecology Department, University Hospital Farhat Hached of Sousse, University of Sousse, Faculty of Medicine of Sousse, Tunisia; 2 University of Monastir, Higher School of Health Sciences and Techniques of Monastir, Tunisia; 3 Department of Family and Community Health, LR12ES03, Faculty of Medicine of Sousse, University of Sousse, Sousse, Tunisia

**Keywords:** infertility, sexuality, ART, FSFI score, female sexuality, sexual function

## Abstract

**Objective:**

To evaluate the impact of infertility and Medically Assisted Procreation (MAP) on female sexuality. Human sexuality presents a multifaceted complexity, shaped by diverse factors and individual intricacies. Infertility and assisted reproductive treatments entail a prolonged and arduous journey, amplifying pre-existing sexual dysfunctions and serving as a rigorous trial of the affected women’s sexuality and the resilience of couples.

**Methods:**

This is a prospective descriptive comparative study with an analytical section, involving 140 female participants: 70 with infertility undergoing assisted reproductive technology (ART) treatment, and 70 who are fertile and had no prior history of conceiving problems. The evaluation of the sexual function of the two groups was conducted using the Female Sexual Function Index (FSFI) score.

**Results:**

The infertile group exhibited a higher level of marital adjustment compared to the fertile cohort. A majority of participants within the infertile cohort (51.4%) presented with primary infertility and (48.6%) experiencing secondary infertility. A marginal elevation in the frequency of sexual intercourse among infertile participants was observed. The mean global score of female sexual function was (21.57±3.36) in the infertile group, which was significantly lower compared to (24.46±1.97) in the fertile group. Notably, both scores fell within the criteria for high risk of sexual dysfunction. The difference between the two groups was significant for all dimensions of FSFI.

**Conclusions:**

Infertility and its treatments pose challenges to female sexuality, often leading to sexual dysfunction. Thus, counseling and sexological support are crucial during treatment.

## INTRODUCTION

A satisfactory sexual experience is characterized by its contribution to the moral and physical well-being of both partners, alongside its potential for reproductive purposes (Touraille & Ågmo, 2024). It is imperative to underscore the absence of an absolute normative framework in matters of human sexuality (Touraille & Ågmo, 2024). Unlike the animal kingdom, where sexual behavior is predominantly instinctual, human sexuality is inherently multifaceted, shaped by linguistic nuances and individual intricacies (Touraille & Ågmo, 2024). However, when a long-awaited pregnancy does not occur immediately, it is common for couples to question their fertility and experience anxiety (Luca *et al.*, 2021). This can create a stressful environment and shift the focus of sexuality towards conception rather than pleasure. The frustration of failure, particularly after a negative pregnancy test, can lead to a loss of interest in sexual activity (Monga *et al.*, 2004; Ohl *et al.*, 2009).

Infertility can cause significant stress and anxiety, leaving couples feeling vulnerable. Moreover, it also, impacts the life of the couples on many aspects such as physically, sexually, psychologically, emotionally and even financially (Monga *et al.*, 2004; Whiteford & Gonzalez, 1995). The focus on sexuality, and the efforts done to obtain a pregnancy, alter the quality of intercourse for the couple. This is caused by the invasive aspect of AMP journey and the absence of spontaneity during sexual activities (Ohl *et al.*, 2009). Furthermore, this situation is likely to exacerbate pre-existing sexual dysfunctions, thereby impeding the couple’s ability to sustain their prior level of sexual activity (Drake & Grunert, 1979).

The couple faces distress and difficulty to express their concerns related to their sexual problems during their consultations, conducing in complicating their sexual dysfunctions (Reder *et al.*, 2009). The process of assisted reproduction is a protracted and arduous journey. Couples who lack the resilience to confront the numerous disappointments, false hopes and extended periods of waiting may emerge from this process either together or separately (Amiri *et al.*, 2015). Hence, the objective of this study is to evaluate the impact of infertility and Medically Assisted Procreation on female sexuality.

## MATERIAL AND METHODS

### Study design and sampling

This is a prospective descriptive comparative study aimed at investigating the impact of infertility and Assisted Reproductive Technology on women’s sexuality with an analytical evaluation, involving 140 female participants: 70 with infertility undergoing assisted reproductive technology (ART) treatment at the ART unit of CHU Farhat Hached Sousse, and 70 who are fertile and had no prior history of conceiving problems, consulting the family planning unit of CHU Taher SfarMahdia for simple complaints. This study spans from December to March 2024. The evaluation of the sexual function of the two groups was conducted using the Female Sexual Function Index (FSFI) score (Giuliano, 2013).This validated questionnaire comprises 19 items assessing sexual activity over the last four weeks. It yields a total score and evaluates six distinct domains of sexual function, including desire, arousal, lubrication, orgasm, sexual satisfaction, and pain. A total score of 26.55 or less indicates sexual dysfunction (Wiegel *et al.*, 2005).

### Inclusion criteria

All the participants in this study were non-menopausal and sexually active for at least four weeks before this assessment. Concerning the infertile women group we included every woman; who consented to participate in the study, aged between 18 to 45 years old, and, undergoing Assisted Reproductive Technology treatment for a period of at least 1 year.

And for the fertile women group, we included every woman; who consented to participate in the study, aged between 18 to 45 years old, and, without medical issues related to reproduction.

### Non-inclusion criteria

We did not include in this study women in their menopause or perimenopause, in a pregnancy, postpartum state or breast feeding, aged more than 45 years, and who were separated from their spouses during the last 2 months

### Exclusion criteria

In this study, we excluded women with vaginismus, congenital malformations, undergoing treatment for psychiatric disorders and women who refused to participate in the study.

## METHODS

The final version of the questionnaire was introduced with a letter outlining the theme and purpose of the research, along with ethical considerations. The questionnaire was completed by a third party, independent of any influence and without a predetermined time constraint. It was distributed by a single operator, trained to explain to the women. The participants who received the questionnaire have basic French language skills. Prior to questionnaire distribution and at the outset of this study, authorization was obtained from the head of the department. Women were informed of the main objectives of the study, as well as the assurance of anonymity and confidentiality of the collected data. Indeed, women were informed that they had the freedom to participate or not in the study, while respecting their choices. Given the potential embarrassment associated with inquiring about sexual related topics within our conservative community, we ensured a comfortable and discreet environment within the hospital for questionnaire completion. Female participants were afforded a separate space to fill out the questionnaires, ensuring privacy. Furthermore, strict confidentiality measures were upheld throughout the process.

### Statistical analysis

A descriptive study was carried out, utilizing means and percentages for qualitative variables, and counts with percentages were determined. The mean and standard deviation for continuous quantitative variables were computed. The Student’s T-test with a significance level of 5% was used to compare means, whereas the chi-square test or Fisher’s exact test was used to compare percentages.

## RESULTS

### Age

In the infertile group, 57.1% of women were aged 30 to 40 years, 24.3% were 40 years or older, and 18.6% were under 30 years old. In contrast, in the fertile group, 55.7% were aged 30 to 40 years, 37.1% were under 30 years old, and only 7.2% were 40 to 45 years old. The difference between the two groups was statistically significant (*p*=0.004).

### Marital adjustment

Half of the participants in the infertile group (51.4%) had an average marital adjustment, while 71.4% in the fertile group had an average marital adjustment.

**Figure 1:**
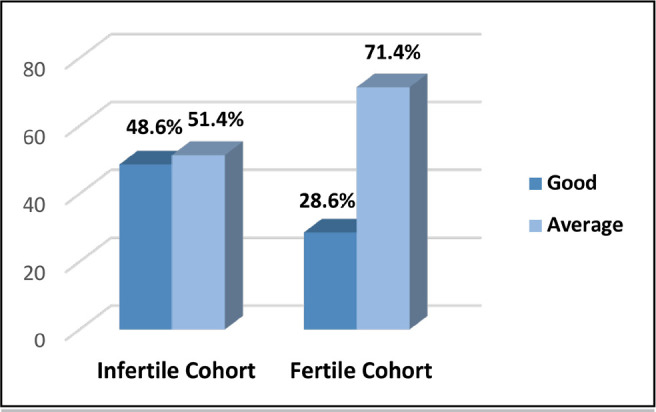
Distribution of participants according to their marital adjustment.

The difference between the two groups was statistically significant (*p*=0.031) (Figure 1).

### Type of infertility

More than half of the participants in the infertile group (51.4%) had primary infertility, while 48.6% had secondary infertility.

### Duration of infertility

The mean duration of infertility in the infertile group was 5.16±3.31 years.

### Origin of the infertility

**Figure 2:**
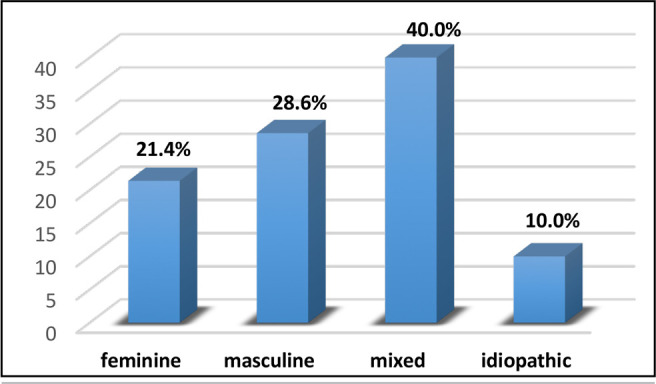
Distribution of participants in the infertile group according to the origin of infertility.

More than 1/3 of the participants (40.0%) had mixed infertility (Figure 2).

### Medically assisted procreation

The mean number of attempts of medically assisted procreation in the infertile group was 2.40±1.12. The mean number of years of infertility treatment in the infertile group was 2.58±1.81 years. More than half of the participants (57.1%) had undergone in vitro fertilization (IVF) (Table 1).

**Table 1:** Distribution of participants in the infertile group according to the medically assisted procreation Unique choice responses.

	Mean±SD	[Min-Max]
Number of attempts of medically assisted procreation	2.40±1.12	[1-6]
Number of years of infertility treatment	2.58±1.81	[1-8]
	**n**	**%**
Type of technique of MAP IVF ICSI IUI	27 15 28	38.6 21.4 40.0

### Sexual life

All participants in both the infertile and fertile groups (100%) reported engaging in sexual intercourse with their husband. In the infertile group, 68.6% engaged in sexual intercourse at least seven times over the past four weeks, however in the fertile group, only 64.3% reported having sexual intercourse seven times or moreover the same period.

Regarding reasons for having sexual intercourse, the most common reasons in the infertile group were for procreation (72.9%), expressing love (80.0%), and maintaining the relationship (85.7%). And the most common reasons in the fertile group were expressing love (95.7%), maintaining the relationship (91.4%) and physiological need (74.3%).

In terms of timing of sexual intercourse, 44.3% of the infertile group reported having sexual intercourse linked to their ovulation period, while 100% of the fertile group had sexual intercourse at any time during their cycle (Table 2).

**Table 2:** Distribution of participants according to their sexual life (n=70).

	Infertile group [n (%)]	Fertile group [n (%)]	p-value
**Do you engage in sexual intercourse with your husband?**			
**No** **Yes**	0 (0.0%) 70 (100.0%)	0 (0.0%) 70 (100.0%)	---
**How often have you engaged in sexual intercourse during the last four weeks?**			
**< 7** **7-14** **> 14**	22 (31.4%) 28 (40.0%) 20 (28.6%)	25 (35.7%) 18 (25.7%) 27 (38.6%)	0.182
**Reasons for having sexual intercourse**			
**Marital obligation**	24 (34.3%)	42 (60.0%)	---
**Procreation**	51 (72.9%)	16 (22.9%)	
**Partner satisfaction**	42 (60.0%)	49 (70.0%)	
**Prevent your spouse from being unfaithful**	3 (4.3%)	9 (12.9%)	
**Expressing love**	56 (80.0%)	67 (95.7%)	
**Physiological need**	42 (60.0%)	52 (74.3%)	
**couple satisfaction**	56 (80.0%)	66 (94.3%)	
**Maintaining the relationship**	60 (85.7%)	64 (91.4%)	
**Is the frequency of your sexual intercourse linked to your ovulation period or do you have sexual intercourse at any time during your cycle?**			
**linked to ovulation period** **any time during cycle**	31 (44.3%) 39 (55.7%)	0 (0.0%) 70 (10.0%)	0.000

### Female sexual function index

The mean score of sexual desire of the infertile cohort was slightly inferior 3.37±0.57 compared to the fertile group’s mean score 3.70±0.49. The infertile group exhibited a lower arousal mean score compared to the fertile group, with scores of 3.46±0.59 and 3.80±0.44 respectively. The mean score of vaginal lubrification in the infertile group was 3.77±0.69, which is significantly lower than the fertile group’s score that is equal to 4.08±0.42. The infertile cohort exhibited a lower mean score of achieving orgasm 3.14±0.97, compared to the fertile participants 3.98±0.42. The difference between the two groups concerning the mean score of sexual satisfaction was statistically significant; with a mean score of satisfaction in the infertile group equal to 4.06±0.84, which is lower than the fertile participants’ mean score 4.55±0.49. A lower mean score of pain during sexual intercourse (Dyspareunia) was observed in the infertile participants 3.80±0.89 compared to the fertile group that exhibited a relatively high mean score 4.35±0.54. The mean global score of female sexual function was significantly lower in the infertile group 21.57±3.36, compared to in the fertile group 24.46±1.97. The difference between the two groups was significant for all dimensions of the FSFI (Table 3).

**Table 3:** Distribution of participants according to their female sexual function index (FSFI) (n=70).

	Infertile group		Fertile group		p-value
	Mean±SD	[Min-Max]	Mean±SD	[Min-Max]	
**Desire**	3.37±0.57	[2.0-5.0]	3.70±0.49	[3.0-5.0]	0.000
**Arousal**	3.46±0.59	[1.8-5.0]	3.80±0.44	[3.0-4.8]	0.000
**Lubrification**	3.77±0.69	[2.0-5.0]	4.08±0.42	[3.3-5.0]	0.002
**Orgasm**	3.14±0.97	[1.0-5.0]	3.98±0.42	[3.0-4.7]	0.000
**Satisfaction**	4.06±0.84	[1.7-5.0]	4.55±0.49	[2.7-5.0]	0.000
**Pain**	3.80±0.89	[1.7-5.0]	4.35±0.54	[3.0-5.0]	0.000
**FSFI-Global**	21.57±3.36	[12.4-27.5]	24.46±1.97	[20.0-28.6]	0.000

## DISCUSSION

### Population’s General Characteristics

The average age of our infertile group falls within the range of 30 to 40 years for 57.1% of women. In contrast, within the fertile group, 55.7% of individuals fell within the age range of 30 to 40 years. In comparison to Reder *et al.* (2009) study, in which he found the majority of his population fell within the age range of 30 and 35 years old. This notable statistical difference can be attributed to various factors, including delayed marriage decisions and socio-economic challenges in Tunisia (Horne, 1992). Our study revealed that the infertile group exhibited a higher level of marital adjustment compared to the fertile cohort. Specifically, 48.6% of the infertile participants demonstrated a favorable conjugal adjustment, whereas only 28.6% of the fertile individuals reported a similarly positive marital adjustment (Figure 1). According to two studies (Eghtedar *et al.*, 2021; Ohl *et al.*, 2009), this phenomenon may be attributed to the challenges posed by infertility and the associated adversities encountered by couples, which potentially fostered greater cohesion and communication, thereby contributing to an improved marital adjustment among infertile couples (Ohl *et al.*, 2009).

### Infertility characteristics

The majority of participants within the infertile cohort 51.4% presented with primary infertility, with the remaining 48.6% experiencing secondary infertility. The mean duration of infertility among individuals in the infertile group was computed to be 5.16±3.308 years. Similar results were found in Benksim *et al*.‘s study (Benksim *et al*., 2018), the primary infertility rates were 67.37% and the secondary infertility rates were 32.63%.

We found that the etiology of infertility exhibited the following distribution, 40% of participants undergoing MAP exhibited mixed infertility, 21.4% attributed their infertility to feminine causes, 28.6% to masculine fertility issues, while the remained was classified as idiopathic in nature (Figure 2). Whereas, Reder *et al.* (2009) findings indicated that idiopathic causes accounted for the primary etiology of infertility, representing 37.2% of cases.

The average number of attempts for medically assisted procreation (MAP) within the infertile cohort in our study was 2.40±1.12, with a mean duration of treatment spanning 2.58±1.81years. The most frequently employed MAP protocol was intrauterine insemination (IUI), accounting for 40.0% of cases, closely followed by in vitro fertilization (IVF), utilized by 38.6% of participants within the infertile cohort (Table 1). In contrast, Reder *et al.* (2009) study revealed a mean duration of MAP treatment ranging between 3 and 4.9 years on average. Moreover, the predominant MAP protocol employed in Reder *et al.* (2009) study was intracytoplasmic sperm injection (ICSI), constituting39.5% of cases.

### Female Sexual Life During Medically Assisted Procreation Treatment

All participants in both groups 100% reported engaging in sexual intercourse with their respective husbands (Table 2). In the infertile group, 68.6% engaged in sexual intercourse at least seven times over the past four weeks, however in the fertile group, only 64.3% reported having sexual intercourse seven times or moreover the same period. Our study revealed a higher frequency of sexual intercourse among the infertile participants compared to the fertile cohort (Table 2). This convergence may be attributed to the heightened emphasis on increasing the likelihood of conception through a greater number of sexual encounters, a practice closely associated with the specific protocol of MAP followed. Notably, patients undergoing IUI displayed a higher frequency of intercourse compared to those undergoing IVF and ICSI procedures (Coëffin-Driol & Giami, 2004; Luca et al., 2021).

Similarly to our study, and according to Reder *et al.* (2009), the frequency of sexual intercourse does not appear to decrease following the treatment of infertility. In the context of coitus, 44.3% of individuals within the infertile cohort reported aligning their sexual intercourses with their ovulation period, aiming to enhance their chances of conception. Notably, the majority of this subgroup were undergoing anIUI protocol. Conversely, the remaining participants demonstrated irregular and non-ovulatory cycle-aligned coital patterns, primarily associated with ICSI or IVF protocols Reder *et al.* (2009) (Table 2). The study of Ohl *et al.* (2009) has shown that the MAP protocol chosen has an impact on the frequency of sexual intercourse, women undergoing IUI are more likely to alginate their coitus schedule with ovulation, increasing the frequency of coitus during that period, unlike women undergoing ICSI (*p*=0.031) or IVF (*p*=0.04). Furthermore, it has been proposed that sexual expression within infertile couples may adopt a *“mechanical and forced”* quality (Wallach *et al.*, 1982). Also in Monga *et al.* (2004) they stated that the infertile women scored higher in sexual frequency and desire compared to the fertile group, and there were no significant statistical differences. However, this apparent disparity is likely an artificial reflection of the perceived need to conceive, rather than an accurate indicator of the health of the sexual relationship within the infertile couple (Monga *et al.*, 2004).

Regarding the motivations for having coitus, we found in our study, that the infertile group claimed that their reasons for intercourse were procreation 72.9% and maintaining the relationship 85.7%, conversely to the fertile group they stated their motivations as expressing love 95.7% and physiological need 74.3%. Monga *et al.* (2004) underscore that when sexuality becomes a mean created to achieve pregnancy, the concerns of the couple may lead one of the partners to excessively demand sexual intercourse, the effects of which could be deleterious. The sexual encounters described herein are deemed “*futile*”, invalidated as they are by the foregrounding pursuit of pregnancy (The impact of Infertility and its treatment on sexual life and marital relationships, review of the literature.pdf, n.d.). This phenomenon is often exacerbated by medical interventions such as post-coital testing, in vitro penetration of cervical mucus during ovulatory periods, ovulation inductions, and prescribing the timing of sexual intercourse, which, despite their intrusiveness into the couple’s intimacy, somewhat legitimize this deviation in sexual behavior (Coëffin-Driol & Giami, 2004). Within the scope of this discussion, attention is directed towards the augmentation in the frequency of sexual intercourse vis-à-vis its qualitative aspect. The decrement in quality is elucidated as a consequence of the diminished spontaneity engendered by the intrusive protocols of Medically Assisted Procreation . On the contrary, Slade *et al.* (1992) found that not only has the quality of intercourse deteriorated, but also the frequency of coitus has decreased significantly (Tayebi & Ardakani, 2009). This compounds the poor sexual and marital adjustment resulting from infertility and its treatments (Slade *et al.*, 1992).

### FSFI Comparison: Female Sexual Function in Fertile and Infertile Women

The classification score FSFI was used as an assessment instrument that addresses the multidimensional nature of female sexual function (Rosen *et al.*, 2000). It was employed to identify potential risk of Female Sexual Dysfunction (FSD)(Table 3). This validated questionnaire comprises 19 items assessing sexual activity over the last four weeks. It yields a total score and evaluates six distinct domains of sexual function, including desire, arousal, lubrication, orgasm, sexual satisfaction, and pain. A total score of 26.55 or less indicates sexual dysfunction (Meston *et al.*, 2020; Wiegel *et al.*, 2005).

#### 1. Desire

The mean score of sexual desire of the infertile cohort was slightly inferior 3.37±0.57 compared to the fertile group’s mean score 3.70±0.49, unlike the Egyptian study (Gabr *et al.*, 2017) results that reported that the mean scores of desire showed no significant difference between the two groups. Other studies (Anderson *et al.*, 2003; Pakpour *et al.*, 2012) insisted on the significant decrease of infertile female’s sexual desire compared to the fertile community. According to Cindy M’s article (Meston *et al.*, 2020) women scoring 5 or lower on the Desire domain likely meet the diagnostic criteria for Hypoactive Sexual Desire Disorder (HSDD), whereas those with scores exceeding 5 are less likely to meet the criteria. However, it is imperative to acknowledge that the FSFI alone does not suffice for diagnosing sexual dysfunction (Meston *et al.*, 2020). Ohl *et al.* (2009) found similar results as our study, showing that the sexual desire and the pursuit of pleasure are diminished due to scheduled coitus, leaving no room for spontaneity, as the primary objective is reproduction. This decrease in sexual desire is evident in practice. It may be apparent that sexual desire appears high due to the frequency of sexual intercourse, which in reality, showcases that sexuality has become more of a tool to conceive rather than a mean of pleasure (Ohl *et al.*, 2009).

#### 2. Arousal

We observed a slight difference between the two groups: the infertile group exhibited a lower arousal mean score value compared to the fertile group, with scores of 3.46±0.59 and 3.80±0.44, respectively. On the contrary, Gabr *et al.* (2017) found no statistical significance between the two groups concerning the mean score of sexual arousal. Conversely, other studies (Tao *et al*., 2011; Audu, 2002; Millheiser *et al.*, 2010; Khademi *et al.*, 2008) supported our results and put an emphasis on the deterioration of infertile female‘s sexual arousal due to infertility and MAP treatments.

#### 3. Lubrification

The mean score of vaginal lubrification in the infertile group was 3.77±0.69, which, is significantly lower than the fertile group’s score that is equal to 4.08±0.42. Khademi *et al.* (2008) insisted in their article that vaginal-dryness and lower vaginal lubrification where more observed in infertile participants undergoing MAP treatments than in the Iranian normal population. However, the study of Gabr *et al.* (2017) reported no significant difference between the two groups concerning vaginal lubrication.

#### 4. Orgasm

The infertile group in our study exhibited a low mean score of achieving orgasm 3.14±0.97, compared to the fertile participants 3.98±0.42. this high-lightens infertility as a risk factor for sexual dysfunction, such as female orgasmic dysfunction FOD(19,26). Supported by the similar results of Gabr *et al.* (2017) study that show a significant difference between the infertile group and the control group (*p* value: 0.01).

#### 5. Satisfaction

The difference between the infertile and the fertile group concerning the mean score of sexual satisfaction was statistically significant (*p* value: 0.001). With a mean score of satisfaction in the infertile group equal to 4.06±0.84, while the fertile participants had a slightly elevated mean score of sexual satisfaction 4.55±0.49, which is relatively close to the maximal score 6.0. These results can be explained by the multiple components of this dimension, and these results are supported by similar findings by Meston *et al.* (2020) and Wiegel *et al.* (2005).

Satisfaction typically pertains to a subjective sense of well-being. In the context of sexual assessment, it can encompass various aspects, including overall satisfaction with the sexual relationship with one’s partner, as well as specific satisfaction related to the partner’s behavior and interactions during sexual activity (Meston *et al.*, 2020; Wiegel *et al.*, 2005). Which can lead participants to evaluate their satisfaction on general sexual well-being with their respective partners. Furthermore, Gabr *et al.* (2017) had similar findings to our study, stating that infertile participants had a lower mean score of sexual satisfaction compared to the control group, equal to 4.9±0.5 and 5.2±0.5, respectively.

#### 6. Pain

Our study showed a lower mean score of pain during sexual intercourse (Dyspareunia) in the infertile participants 3.80±0.89 compared to the fertile group that exhibited a relatively high mean score of dyspareunia 4.35±0.54. These results may be explained by the parity difference between the two groups. Since the fertile group had a 100% rate of previous childbirth, particularly in cases of antecedents vaginal delivery, the prevalence of dyspareunia had augmented (Fauconnier *et al.*, 2012). Similar results were found in the study of Gabr *et al.* (2017), stating that there was a significant difference between the infertile and the control group concerning the mean score of dyspareunia, 3.9±0.9 and 4.4±0.7, respectively.

#### 7. FSFI global score assessment

The mean global score of female sexual function was 21.57±3.36 in the infertile group, which was significantly lower compared to 24.46±1.97 in the fertile group. Notably, both scores fell within the criteria for high risk of sexual dysfunction, as they were below the threshold of the global score of 26.55. However, a marked difference was observed between the minimal and maximal scores in both groups. The minimal global score was (12.4), and the maximal score was 27.5. Interestingly, the fertile group exhibited a higher minimal score of 20.0 compared to the infertile group. Furthermore, the fertile group demonstrated a significantly higher maximal total score of 28.6 compared to the infertile group. To further elucidate, despite the fertile group exhibiting a higher global FSFI score compared to the infertile group, it still fell into the high-risk category for Sexual Dysfunction (SDD). This outcome may be attributed to poor marital adjustment within the fertile cohort, leading to misunderstandings and miscommunications between partners. In Tunisian marriage culture, the extended family often influences the couple’s intimate life, creating a paradoxical effect where sexuality is treated with caution and considered taboo. This cultural dynamic may consequently worsen their sexual well-being (Frini & Muller, 2023). Similar results were found in the study conducted by Mirblouk *et al.* (2016). However, in an Egyptian study done by Gabr *et al.* (2017) they found different results compared to us, 26.8±3.8 as a mean global score for the infertile group while the fertile group exhibited a mean global score equal to 27.9±3.5. This difference in results between this study and ours can be explained by the difference in the size of the population chosen. Comparable results to those observed in our study, regarding the lower mean global score of FSFI in the infertile group compared to the fertile group, were documented in the research conducted by Pakpour *et al.* (2012). The difference between the two groups, in our study, was significant for all dimensions of FSFI, however in Gabr *et al.* (2017) they stated that the total FSFI scores, as well as the scores for the orgasm, satisfaction, and pain sexual domains, were significantly lower in the infertile group. In contrast, the desire, arousal, and lubrication scores did not show significant differences between the groups (Gabr *et al.*, 2017). This indicates that the orgasm, satisfaction, and pain sexual domains are more affected in infertile women compared to other sexual domains (Gabr *et al.*, 2017).

## CONCLUSION

Through this study, we can ascertain that infertility and its treatments at Assisted Procreation Technology (APT) centers exert a substantial influence on female sexuality. Our observations showed that despite the increased frequency of sexual encounters among infertile women and their respective husbands, sexuality often transitions from a source of pleasure to a mean of conception, thereby diminishing its intrinsic enjoyment and transforming it into a mere tool utilized in pursuit of motherhood.

Furthermore, a notable disparity in sexual function was observed between the two groups. Our infertile cohort exhibited significantly lower FSFI scores across multiple dimensions of sexuality compared to the fertile group, including sexual desire, arousal, lubrication, orgasm, and sexual satisfaction. Consequently, these findings place infertile participants at a heightened risk for female sexual dysfunction. It is imperative to recognize that sexual dysfunction can both contribute to fertility issues and emerge as a consequence of the diagnosis of couple infertility. Additionally, it is crucial to acknowledge the stigma experienced by many patients stemming from their inability to conceive or from unsuccessful attempts at Medical Assisted Procreation (MAP), thereby exacerbating the challenges faced by those undergoing infertility treatments. Thus, women are more affected by this medical issue, or at least they report it more often. However, this study only scratches the surface of the iceberg; undoubtedly, many unspoken issues remain unresolved. Nevertheless, it has the merit of being a starting point to initiate dialogue within the couple about their sexuality.
